# Controlling Geminiviruses before Transmission: Prospects

**DOI:** 10.3390/plants9111556

**Published:** 2020-11-12

**Authors:** Muhammad Salman Mubarik, Sultan Habibullah Khan, Aftab Ahmad, Ali Raza, Zulqurnain Khan, Muhammad Sajjad, Reda Helmy Ahmed Sammour, Abd El-Zaher M.A. Mustafa, Abdullah Ahmed Al-Ghamdi, Amal H. Alajmi, Fatin K. I. Alshamasi, Mohamed Soliman Elshikh

**Affiliations:** 1 Centre for Agricultural Biochemistry and Biotechnology (CABB), University of Agriculture, Faisalabad 38040, Pakistan; sultan@uaf.edu.pk; 2 Center of Advanced Studies in Agriculture and Food Security (CAS-AFS), University of Agriculture, Faisalabad 38040, Pakistan; aftab.ahmad@uaf.edu.pk; 3 Department of Biochemistry, University of Agriculture, Faisalabad 38040, Pakistan; 4 Key Lab of Biology and Genetic Improvement of Oil Crops, Oil Crops Research Institute, Chinese Academy of Agricultural Sciences (CAAS), Wuhan 430062, China; alirazamughal143@gmail.com; 5 Institute of Plant Breeding and Biotechnology (IPBB), MNS University of Agriculture, Multan 66000, Pakistan; zulqurnain.khan@mnsuam.edu.pk; 6 Department of Biosciences, COMSATS University Islamabad (CUI), Park Road, Islamabad 45550, Pakistan; msajjadpbg@gmail.com; 7 Department of Botany and Microbiology, College of Sciences, King Saud University, P.O. Box 22452, Riyadh 11495, Saudi Arabia; rsammour@ksu.edu.sa (R.H.A.S.); abdalghamdi@ksu.edu.sa (A.A.A.-G.); amal.h.alajmi@gmail.com (A.H.A.); 0111718192@yahoo.com (F.K.I.A.); melshikh@ksu.edu.sa (M.S.E.); 8 Botany Department, Faculty of Science, Tanta University, Tanta 31511, Egypt

**Keywords:** geminiviruses, genetic engineering, genetically modified (GM) crops, whitefly, virus transmission

## Abstract

Whitefly (*Bemisia tabaci*)-transmitted Geminiviruses cause serious diseases of crop plants in tropical and sub-tropical regions. Plants, animals, and their microbial symbionts have evolved complex ways to interact with each other that impact their life cycles. Blocking virus transmission by altering the biology of vector species, such as the whitefly, can be a potential approach to manage these devastating diseases. Virus transmission by insect vectors to plant hosts often involves bacterial endosymbionts. Molecular chaperonins of bacterial endosymbionts bind with virus particles and have a key role in the transmission of Geminiviruses. Hence, devising new approaches to obstruct virus transmission by manipulating bacterial endosymbionts before infection opens new avenues for viral disease control. The exploitation of bacterial endosymbiont within the insect vector would disrupt interactions among viruses, insects, and their bacterial endosymbionts. The study of this cooperating web could potentially decrease virus transmission and possibly represent an effective solution to control viral diseases in crop plants.

## 1. Geminiviruses: Impact on Crop Plants

Geminiviruses are transmitted by an insect vector, sweet potato whitefly (*Bemesia tabacci*) [1]. In tropical and subtropical regions of the world, whitefly infestation has caused devastating disease outbreaks and, consequently, a massive reduction in crop yields [2]. The occurrence of whitefly-transmitted viruses in agriculture presents a huge challenge for researchers concerned with yield and quality in plants [3]. Geminiviruses infection leads to the development of several undesirable symptoms in plants, such as leaf curling, yellow discoloration, stunted growth, and reduced yield [4,5]. It is difficult to find a field without a virus infection over several kilometers once whiteflies have transferred the virus into several plants.

## 2. Whitefly: Vectoring Geminiviruses

Insects are an essential group of vectors for the spread of various plant viruses. Most plant viruses rely on insect vectors for their persistence and spread [6,7]. It is estimated that over 75% of plant virus species are vectored by insects [8]. Despite having the established knowledge about virus transmission by plant cell sap, little is known about the mechanisms and requirements for insect vectors.

The association between insect vectors and plant viruses is quite intricate and understood for all viruses and insect species. The mouthparts of most sucking insects are highly adapted for the transmission of viruses into plants [9]. Successful virus transmission starts with the ingestion of virus particles from infected plants and ends with their transfer to healthy plants. However, the transmission of virus particles is the result of a multifaceted interaction among the host, vector, and pathogen [10,11].

Whitefly, as a vector, has a persistent mode of transmission and widespread host range of 74 plant families with over 500 different plant species [12]. Whitefly is the insect vector of different virus families, including *Geminiviridae* and *Potyviridae*. Whiteflies are of major agricultural concern because they can cause detrimental effects to plants simply from feeding [13]. In addition to feeding, whitefly is also known as a vector for 100 Geminiviruses and other plant viruses [14]. Major crops infected by whitefly-transmitted Geminiviruses include cotton, common bean, mungbean, blackgram, lima bean, cowpea, soybean, tomato, potato, eggplant, pepper, chilli pepper, melon, watermelon, squash, okra, and cassava [15]. 

Whitefly contains endosymbiotic bacteria situated inside specialized host cells [16]. The association between whitefly and bacterial endosymbionts is a consequence of a single infection of the host, followed by endosymbionts transmission to the whitefly offspring [17]. The endosymbionts of whitefly and other insect vector hosts play a key role in virus transmission [18,19]. Whitefly and their bacterial endosymbionts commonly form close symbiotic associations that emerge from co-evolution. All whitefly species comprise a primary endosymbiotic bacterium, *Portiera aleyrodidarum* [20]. In addition to *P. aleyrodidarum*, the whitefly species contain one or more additional secondary endosymbionts, including *Arsenophonus*, *Cardinium*, *Fritschea*, *Hamiltonella*, *Rickettsia*, and *Wolbachia* [21,22,23]. *Buchnera aphidicola*, the primary endosymbiont of aphids, secretes copious amount of a single 60 kDa protein, named “symbionin” [24], known later as the GroEL chaperonin, and it can comprise up to 10% of cellular proteins of *B. aphidicola* [25,26]. 

**Figure 1:**
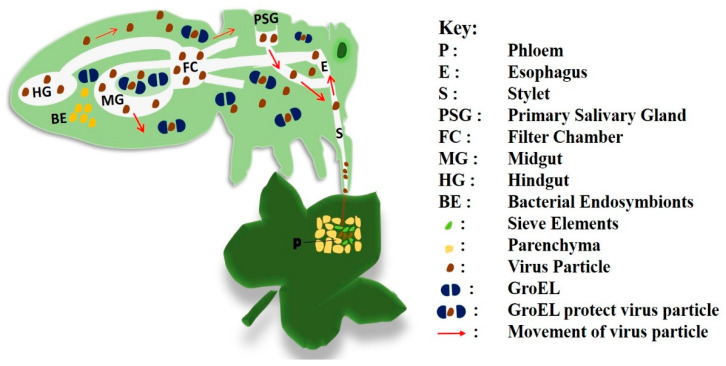
The transmission cycle starts with virus acquisition from the phloem of an infected plant. The virus particles (brown) move along the stylet, foregut, and esophagus and reach the midgut in whiteflies. GroEL (blue) is required for protection (blue-brown-blue), circulation, and transmission of virus particles.

GroEL, a heat shock protein, is a key factor in the circulative transmission of Geminiviruses [27,28]. It is a heptameric cylindrical protein that usually forms a complex with a smaller protein, called GroES [29]. In the virus transmission cycle, GroEL binds to the virus coat protein in the body cavity (hemocoel) of an insect circulatory system that contains blood and then chaperones to the salivary glands for the completion of virus circulation [30,31]. The GroEL symbionin of green peach aphid (*Myzus persicae*) is known to bind to the coat protein of a luteovirus and protect virus particles from rapid proteolysis in the gut and hemolymph of the aphids [32]. Overexpression of GroEL protein seems to be an important feature of bacterial endosymbionts in insects. Once the GroEL escorts the virus through the whitefly’s hemocoel to its salivary glands, next time the whitefly feeds, the virus is transferred to the new plant from the whitefly’s saliva [7,33]. This unique function of GroEL is a necessary step for the successful transmission of the virus to a new host plant (Figure 1) [34,35].

## 3. Difficulties in Achieving Resistance against Geminiviruses 

Existing strategies to control virustransmitted plant diseases mainly focus on managing either the insect vector or the virus itself, rather than the interaction between them [36]. It is hypothetically easier to target vector populations or viruses after host infection; for most of these strategies, very little information is available about insect vector and plant virus interfaces. 

Traditional approaches to controlling plant virus infection and spread usually involve controlling the whitefly population using various insecticides [37]. However, this approach is not effective or reliable because the whitefly has already transmitted the virus, and it may be too late to control the infection in plants. Moreover, insecticides cause acute toxicity to other living organisms and have adverse effects on the environment [38].

Another possible control for viral pathogens is chemical treatments that degrade the viral coat protein in infected plants to prevent the spread of infection by the vector [39]. However, the development of an effective chemical is costly and time-consuming because it must be safe for crops and human contact and/or consumption. It may also be possible to engineer plants using RNA interference (RNAi) to destroy the RNA of virus coat protein upon detection and before infection [40]. However, it is not feasible to manipulate the coat protein of all virus molecules. It is practically challenging to transform plants with multiple RNAi constructs to engineer a broad-spectrum virus resistance by targeting the coat protein of different viruses. Moreover, this is not a practical solution to address the agricultural problems posed by virus transmission.

Various transgene strategies have previously been adopted to control viruses or their vectors. Pathogen-derived resistance, with and without protein expression, has successfully been applied in various crop plants [41,42]. Cuticular waxes and exploitation of natural defense mechanisms against insect vectors act as physical barriers by making the insects attachment to the plant surface difficult and hindering insect movement [43,44]. However, several countries deregulated the cultivation of these transgenic plants [45]. Hence, it is plausible to genetically modify (GM) some vulnerable crops to minimize the effects of infection. While this would take substantial testing to ensure the safety and healthfulness of GM crops, it would be worthwhile to decrease this pathogen problem by using other non-GM approaches.

Advances in microbiome studies have brought attention to the influence of microbial proteins in the virus transmission cycle. The use of genetically engineered microbial genes to alter the vector’s capacity of transmitting virus particles could possibly provide a pragmatic solution. The transmission efficiency of the virus to its host is determined by molecular interactions between GroEL homologues and plant viruses [46], indicating that disruption in such interactions may block the transmission of the virus. Because of the specificity of virus transmission by insect vectors, there are defined stepladders that represent good targets for control strategies to stop the disease cycle.

Previous studies on plant virus transmission mechanisms revealed that insect endosymbiotic GroEL chaperonins play a vital role in the transmission of different plant viruses, including tomato yellow leaf curl virus (TYLCV) [30] and potato leafroll virus (PLRV) [47]. GroEL protein of *Buchnera* found in the aphid hemolymph is considered a critical protein in the transmission of PLRV and barley/cereal yellow dwarf virus (B/CYDV). It was also reported that aphids and whiteflies, fed on anti-*Buchnera* GroEL antiserum, have reduced transmission efficiency of PLRV and TYLCV, respectively [34]. Aphid-transmitted PLRV interacts with GroEL homologues of bacterial endosymbionts to avoid destruction in the insect vector’s haemolymph [47]. Similarly, in whitefly, *Hamiltonella* endosymbiont-secreted GroEL homologues interact with the coat protein of TYLCV [48]. Transgenic plants that overexpress the bacterial GroEL protein found in insects stave off virus disease symptoms despite infection [49,50]. A 30-fold decrease in virus transmission efficacy in whitefly was also achieved by altering the interaction between endosymbiotic GroEL protein and virus coat protein [51].

Moreover, members of the virus genera begomovirus, tospovirus, and luteovirus bind with GroEL protein, indicating that it possibly traps these viruses [52]. The role of GroEL in virus transmission has been verified by well-characterized biochemical properties of GroEL protein [7]. The ostensible specificity of these interactions and virus transmissions opens new avenues for meddling, interference, and control of virus transmission and vector populations. Thus, it is more practical to control the virus within the vector species using the known binding capabilities of bacterial GroEL protein with virus molecules.

## 4. Stopover Geminiviruses Transmission: Prospects

Some genes “drive” themselves through populations by inflating their odds of being inherited. Examples include mobile genetic elements that insert copies of themselves elsewhere in the genome [53], selfish endonucleases [54], and heritable microbes, such as *Wolbachia*, a natural bacterium present in up to 60% of insect species, including some mosquitoes [55,56]. Recently, in Rio de Janeiro, Brazil, and Medellin, Colombia, there were wide releases of mosquitoes that carry *Wolbachia*, which hinders the insects’ ability to transmit Zika, dengue, and other viruses [57]. These bacteria influence the sex of their vector’s offspring and can hinder their fertility. They can also block viruses from reproducing in infected fruit flies and mosquitoes [58]. Although gene drive has not been applied for the prevention of plant virus transmission by targeting the bacterial endosymbionts and genes essential for sex determination of insect vectors, the gene drive system can be engineered to impede the ability of the insect vectors to acquire and transmit viruses.

Bacterial endosymbionts play a vital role in insect nutritional ecology by providing supplemental nutrients, which the host-vector cannot obtain directly from its restricted diet of plant phloem, and aiding in food digestion [59,60]. These bacterial endosymbionts also facilitate their host by protecting against pathogens and reducing susceptibility to pesticides [61,62]. The insect mutualists and their novel interactions have resulted in invasions of new and more virulent insect pests. Conversely, host-specific bacterial endosymbionts can be exploited to advance pest control strategies and discover more about damaging insect species and their microbial associates.

Various transgenic approaches used in crop plants with the aim to target plant viruses are not durable or need extensive biosafety controls [63]. The ultimate cost of whitefly management practices using insecticides affects the country’s economy [64]. If the target of virus control measures would be whitefly or its bacterial endosymbionts, there will be fewer chances of developing resistance in the viruses itself. It could be possible by knocking-out/down of the GroEL gene of the whitefly bacterial endosymbionts and interfering with virus transmission. Additionally, engineered bacterial endosymbionts could play a vital role in developing disease resistance, as well as insect/pest control strategies.

A general method for assuring engineered traits would be favored by natural selection so that the targeted traits could spread over generations to their wild types. This capability would allow us to address various agricultural issues: the rise of pesticide resistance and the damage caused by invasive insect/pest species to agriculture and the environment, including the spread of insect-borne diseases.

Genetic manipulation of bacterial endosymbionts is a well-suited multifaceted strategy; it could potentially control insect/pest transmitted viral diseases to improve agriculture production compared to other pest control interventions. A compelling solution to stop virus transmission through whitefly in plants would be to halt the production of bacterial GroEL protein in the insect’s hemocoel. GroEL homologues are present in *Portiera* (Accession No. WP_014895132), *Hamiltonella* (Accession No. WP_016857397), and *Rickettsia* (Accession No. AJD80658) endosymbionts of whitefly and probably interact with viral particles, owing to their high sequence conservation. In addition to the endosymbiont *Hamiltonella*, a member of the genus Arsenophonus also transmits cotton leaf curl virus (CLCuV) [65]. Additionally, the whitefly GroEL protein is 80% similar to the aphid GroEL protein and has similar involvement in TYLCV transmission [28]. The N-terminal and C-terminal of GroEL protein have a capacity to interact with luteoviruses [66]; these regions are evolutionarily conserved among the *Buchnera* GroEL homologues proteins that can bind several luteoviruses [67]. Moreover, the high amino acid similarity at N-terminal between whitefly and aphid GroEL proteins suggested that at least this region of the protein has been conserved during evolution. Therefore, despite their host, GroEL protein from all bacterial endosymbionts could interact with viral particles and protect them from proteolysis. A possible strategy is to engineer these endosymbiotic bacteria of whitefly producing double stranded RNA (dsRNA) homologous with a GroEL gene for optimal post-transcriptional gene silencing (PTGS). Additionally, with Clustered Regularly Interspaced Short Palindromic Sequences/CRISPR associated-9 CRISPR/Cas9, mediated genetic manipulation of targeted genes and their distribution among targeted populations (insect/pest, plants) has become more robust and swifter than simple genetic inheritance [68]. Moreover, in recent years, GroEL proteins have gained growing attention, as revealed by its startling roles, partly unrelated to chaperone function. It appears to be a key player in sustaining long-lasting mutualistic interactions between bacteria and their hosts. However, very little is known about structural and functional relationships of GroEL homologues undergoing activities, besides the well-characterized chaperone function. It has become evident that GroEL proteins possess diverse functions, specifically in mutualistic interactions of bacteria and insects, essential symbiosis factors in bacteria-insect interactions, the target of the insect immune system, a virulence factor of the entomopathogenic factor, and, more importantly, a transmission factor for plant viruses in insect vectors [51]. Therefore, knocking-out/down of the GroEL gene will potentially block the virus transmission and could impact the insect nutritional pattern that eventually helps to mitigate the plant damage triggered by whitefly infestation.

**Figure 2:**
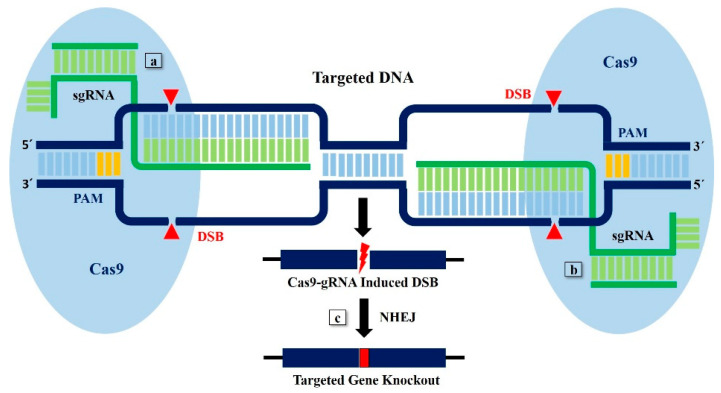
The specificity and wide adaptability of the CRISPR-Cas9 system offer huge potential for GroEL gene knockout. (**a**,**b**) At its simplest, the CRISPR-Cas9 system consists of the chimeric gRNA, which guides the Cas9 endonuclease to the target site. The target site of the GroEL gene is comprised of 20-bp of homology with the gRNA and a protospacer adjacent motif (PAM) sequence. Gene knockout is based on Cas9 endonuclease activity. A pair of Cas9 cleaves the target DNA and creates double-strand breaks (DSBs) at two different sites, which results in excision of genomic DNA fragments of the GroEL gene. (**c**) An imprecise non-homologous end joining (NHEJ) mediated repair will join the remaining DNA fragments.

**Figure 3:**
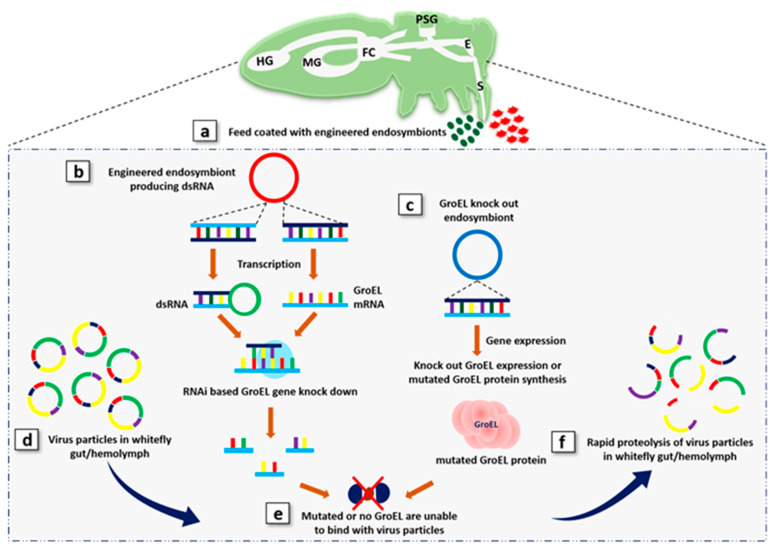
A proposed strategy to knockdown virus transmission by whitefly with the CRISPRCas9 system and RNA interference (RNAi) approach. (**a**) Feed coated with dsRNA, producing engineered bacterial endosymbionts (green particles) and the knockout/mutated GroEL gene, containing engineered bacterial endosymbiont (blue particles). (**b**) Whitefly ingesting engineered bacterial endosymbionts expressing dsRNA to evoke the RNAi mechanism to knockdown the GroEL mRNA produced by endosymbiotic bacteria. (**c**) Whitefly-feasting engineered bacterial endosymbionts with GroEL knockout or mutant protein expression. (**d**) Virus particles in whitefly gut/hemolymph from infected plants. (**e**) Mutated GroEL protein, which is unable to protect virus particles. (**f**) Virus particles would be rapidly degraded by proteolysis in the gut/hemolymph by the whitefly immune system.

Researchers have made significant progress in understanding the genome and how it could be engineered using gene drive technology. Much of the recent attention has been given to the development of the CRISPR-Cas9 system, mainly because of its exceptional versatility as a gene editor in sexually reproducing organisms. Even though a fitness cost or a “selective disadvantage” is usually introduced by gene editing, the increased inheritance rate reflected in CRISPR-Cas9 still enables edited genes to spread, even when the inserted gene’s fitness cost is higher [69]. There is no selective pressure to retain a functioning endonuclease for the suppression drive fixed in a target population, as in the case of the GroEL gene, so that the CRISPR-Cas9 cassette will gradually accumulate mutations [70]. These mutations have normal Mendelian inheritance and can therefore be spreaded through genetic drift in a target population. This could experimentally be achieved by designing guide RNA (gRNA), targeting the GroEL gene of bacterial endosymbionts (Figure 2). Therefore, constitutive delivery of feed, coated with engineered endosymbiotic bacteria, and their transmission would have detrimental effects on the integrity of virus particles in whitefly (Figure 3). Stalling GroEL production in whiteflies would interrupt the virus transmission cycle by preventing vectoring by the whitefly. Targeting GroEL gene expression in the whitefly would also create a transmissible knockdown phenotype. Genetically engineered bacterial endosymbionts-mediated control of virus transmission can be more attractive, since it allows indirect manipulation of the pest species, as it is more complicated to target the pest species with classical genetic engineering innovations directly. Moreover, horizontal gene transfers are also reported in parasites, pathogens, and endosymbionts [71]. A comprehensive study of community structure and genetic variability in whitefly endosymbionts clearly indicate the horizontal transfer, as suggested by proximity between mitotypes sufficient for gene flow at overlapping mitotype ecological niches [72]. Therefore, endosymbiotic bacteria carrying the knockout GroEL gene could horizontally transfer to other related endosymbiotic populations [70]. Consequently, it is anticipated that these strategies can halt virus transmission by their insect vectors at the notch in a few years.

These strategies could offer a feasible potential biocide to control insect populations, using synthetic biology approaches and engineered endosymbiotic bacteria with the knockout GroEL gene, producing dsRNA targeting GroEL gene expression. This allows the development of insect lines, in which systemic RNAi knocks down the expression of the targeted gene without the trauma of alternative RNAi delivery methods [73]. Constitutive expression of dsRNA in whitefly permits a sustainable RNAi mechanism so that phenotypic variations related to loss of function at each developmental phase can be evaluated. In addition to vertical transmission of bacterial endosymbionts, its DNA can be naturally transferred from one species to another through horizontal gene transfer. Additionally, these strategies provide a potential means for highly specific and targeted biocontrol against viral diseases and agricultural pest species. It may control the virus load in whitefly gut and hemolymph and its transmission into the plant cell sap, which would result in suppression of virus infection in plants. This would halt the transmission of viruses and decrease the fitness of a huge herbivore pests on many agricultural plants.

## 5. Conclusions

Opportunities for future research abound, including the development of antiviral treatments. The identification of inimitable steps in the viral infection process, which will possibly restrict the infection process, is a propitious slant for the control of the viral disease. The blooming of advanced biotechnology-based approaches, including RNAi and CRISPR/Cas9, can be a foremost stride toward restricting virus transmission by targeting pathways in the transmission cycle. In insect vectors, expression of exogenous dsRNA is often limited by delivery methods, which restrict the development of RNAi based biocides and reverse genetics studies. A culturable, symbiont-mediated RNAi approach would serve as a powerful tool to integrate gene function into invertebrates. This transformative technology has already been practiced in two evolutionarily distinct insect species (*Rhodnius prolixus* and *Frankliniella occidentalis*) [73]. Both model and non-model insect/pest species can potentially be targeted for genetic manipulation of bacterial endosymbionts in the future. However, the endosymbiont-mediated RNAi approach is anticipated to be effective in the number of insect species. The fundamental requirement is that insects harbor cultivable endosymbionts, a criterion that many globally important insects’ species, including whitefly, already know to meet [74]. The cultivation of endosymbionts mentioned in this article is currently not reported. Still, other whitefly endosymbionts have been cultivated and will provide an opportunity to translate these outcomes into these endosymbionts. In addition, this approach also offers a potential means of targeted biocontrol against various disease vector species. The widespread implications of RNA-guided genome engineering in agriculture and medicine demand a thoughtful and integrated response.

Until now, few proof-of-concept studies have demonstrated the use of the CRISPR/Cas9 system to eliminate certain genes in the fruit fly (*Drosophila melanogaster*), two mosquito species (*Anopheles stephensi* and *Anopheles gambiae*), and in yeast (*Saccharomyces cerevisiae*) [75,76,77,78]. Currently, these cases are interminable and are likely to be released in the upcoming years. Different types of risks associated with these reports should be addressed, including resistance to CRISPR/Cas9 gene cassette when Cas9 endonuclease is unable to recognize and cleave the targeted DNA owing to evolution by de novo mutation and altered mating preferences due to behavioral changes. Therefore, the individual, holding a partial copy of the cassette, inflicts significant fitness costs that can prevent such mutations from spreading. For different gene drive scenarios, researchers could classify candidate genes for gene drives, evaluate gene flow between target and non-target populations or organisms using population genomics approaches, and customize demographic models. Eventually, these contributions would assist stakeholders, legislators, and community members in making informed decisions about the use and regulation of these gene drives.

Numerous other practical difficulties must be overcome before gene knockout becomes a common genetic intervention. However, rapid scientific progress on the CRISPR/Cas9 system and the various outcomes accessible through gene knockouts suggests that biologists will succeed to alter the genomes of wild type pests by providing targeted population suppression. There are convincing opinions in favor of targeting bacterial endosymbionts to eradicate insect-borne viral diseases in crop plants. The evolution of insect resistance to pesticides is a huge drawback for agriculture. Presumably, resistant populations will remain resistant in the absence of pesticides, unless the relevant alleles impose a substantial fitness cost.
